# Oral erythema multiforme related to bronchodilators intake: A report of a case

**DOI:** 10.1002/ccr3.6331

**Published:** 2022-09-14

**Authors:** Maroua Garma, Sameh Sioud, Mounir Omami, Afef Slim, Chokri Abdellatif, Jamil Selmi

**Affiliations:** ^1^ Department of Oral Medicine and Oral Surgery University Clinic of Dental Medicine, University of Monastir Monastir Tunisia; ^2^ Faculty of Dental Medicine, Laboratory of Oral Health and Maxillofacial Rehabilitation (LR12ES11) University of Monastir Monastir Tunisia

**Keywords:** drug, erythema multiforme, oral mucosa, reaction

## Abstract

Erythema multiforme is a reactive inflammatory mucocutaneous disorder. It is classified into three groups: erythema multiforme minor, major, and oral erythema multiforme. The oral mucosa is mostly involved. The etiology of this lesion varied from bacterial, viral, or fungal infection to drug reaction. The aim was to report a case of oral erythema multiforme related to drug intake, in order to highlight clinical and histological features in addition to therapeutic modalities of drug‐induced oral erythema multiforme. A 74‐year‐old female patient consulted for painful ulcerations in the oral cavity, associated with burning sensation and inability to eat or swallow for the past 8 days. After detailed anamnesis and clinical examination, the diagnosis of oral erythema multiforme related to drug intake was retained. Oral erythema multiforme is a benign inflammatory disorder involving the oral mucosa with pathognomonic clinical and histological features. It may result from viral, bacterial, or fungal infection and from drug intake reaction. The interruption of the implicated molecule associated with symptomatic treatment is the principal therapeutic modalities.

## 1 INTRODUCTION

Erythema multiforme is an acute inflammatory mucocutaneous disorder, that can affect skin or mucous membrane or both. It is classified into three groups based on the severity and the number of mucosal sites involved.^1^, ^2^ The first is erythema multiforme minor if ulceration involved a single mucosal site with typical skin target lesions. The second group is erythema multiforme major when lesions involve more than one mucous membrane with skin target lesions. Third, a new category is described in case of absence of skin lesion called oral erythema multiforme. The oral mucosa is the most commonly affected mucous membrane.^1^ Oral manifestations of erythema multiforme are very frequent, and they represent more than 70% of cases of erythema multiforme.^1^, ^3^, ^4^

A case of erythema multiforme resulting from drug intake was reported, in order to highlight the close link between erythema multiforme and medicines consumption and detail the etiology, the clinical features, and the therapeutics modalities of this disease.

## 2 CASE REPORT

A 74‐year‐old female patient consulted with the chief complain of painful ulcerations in the oral cavity associated with burning sensation and inability to eat or swallow for the past 8 days.

She was hypertensive treated by captopril®.

She had a bronchial asthma for which she took every day theophylline® 100 mg for many years. She gave a history of asthma attack 2 weeks back; for this reason, a broncho dilatator was prescribed: Salbutamol® 2 mg. Within 3 days, she developed this oral lesion.

**FIGURE 1:**
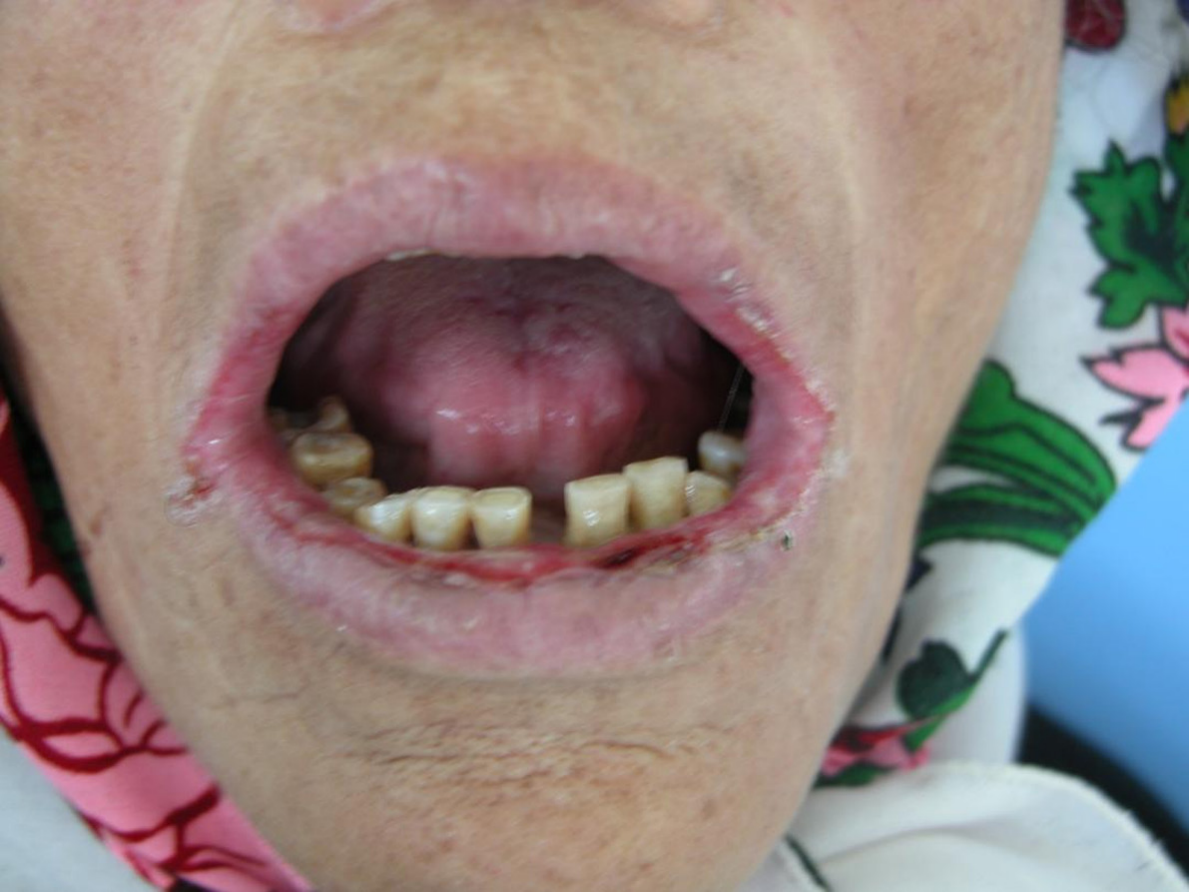
Ulcerations, cracking and fissuring with hemorrhagic crusts on the upper and lower lip

The extra‐oral examination showed extensive irregular ulcerations, cracking, and fissuring with bloody crusting on the upper and lower lip (Figure 1).

**FIGURE 2:**
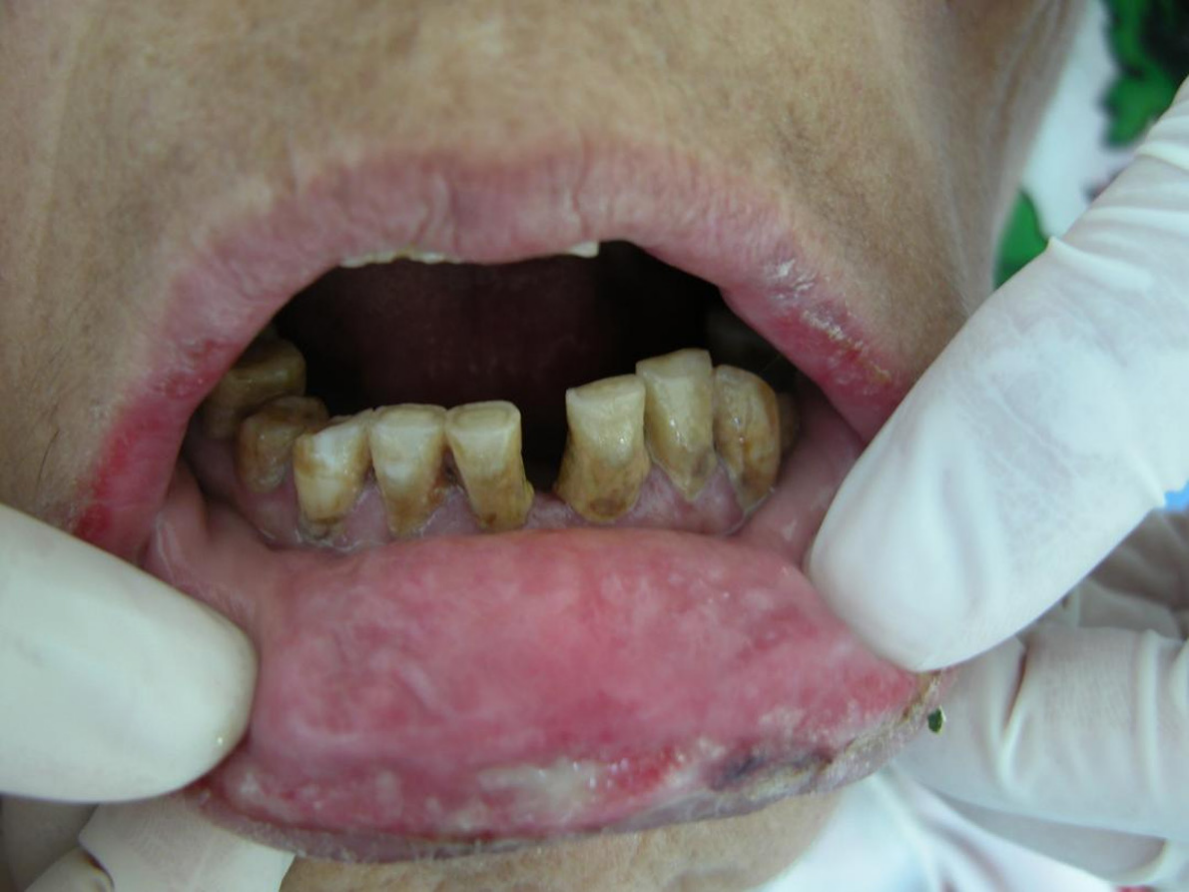
Extensive irregular ulcerations with erythematous borders and some erosions in the buccal surface of lips

**FIGURE 3:**
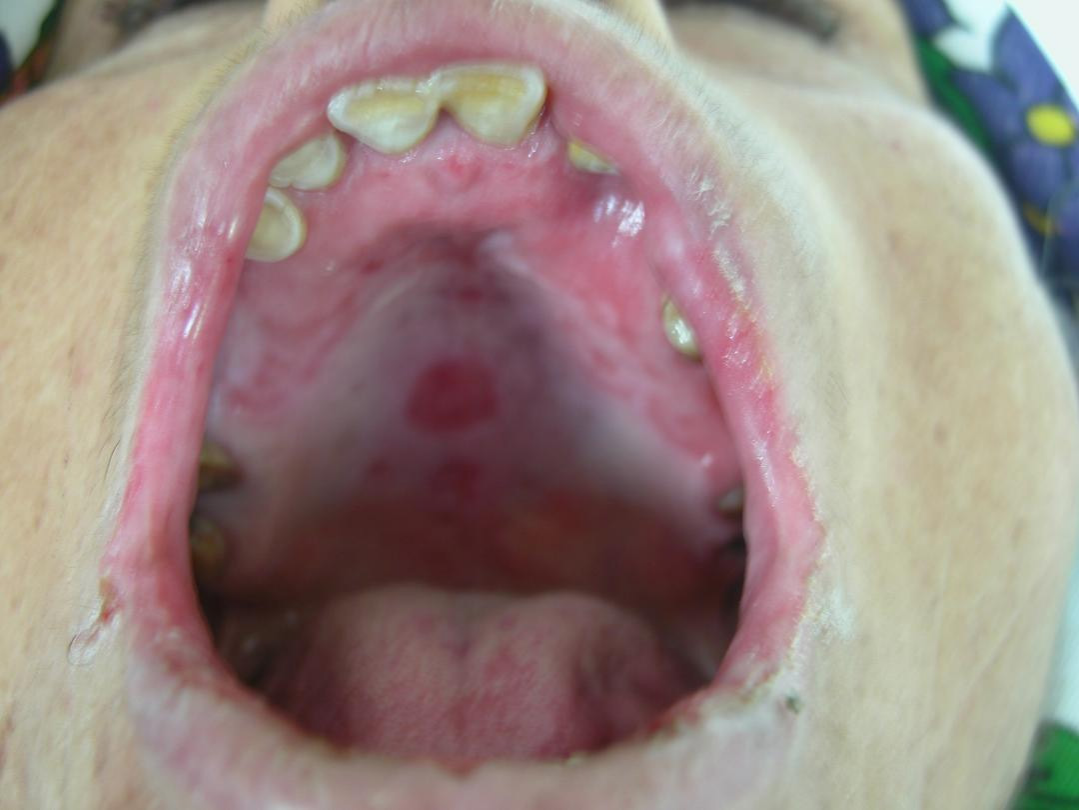
Erosion and white coating revetment in the palate

The intra‐oral examination revealed extensive irregular ulcerations with erythematous borders, some erosions that correspond to broken blisters, in the buccal surface of lips, hard palate, right crest, and dorsal surface of the tongue, in addition to a white coating revetment in the palate (Figures 2 and 3).

The coincidence between the incidence of the oral lesions and drug intake, besides the pathognomonic clinical appearance of the ulcerations, was in favor of the diagnosis of drug‐induced erythema multiforme.

The patient was advised to interrupt her recent medication: Salbutamol® 2 mg with the agreement of his doctor who was recommended to substitute the molecule. A topical corticosteroid was prescribed.

**FIGURE 4:**
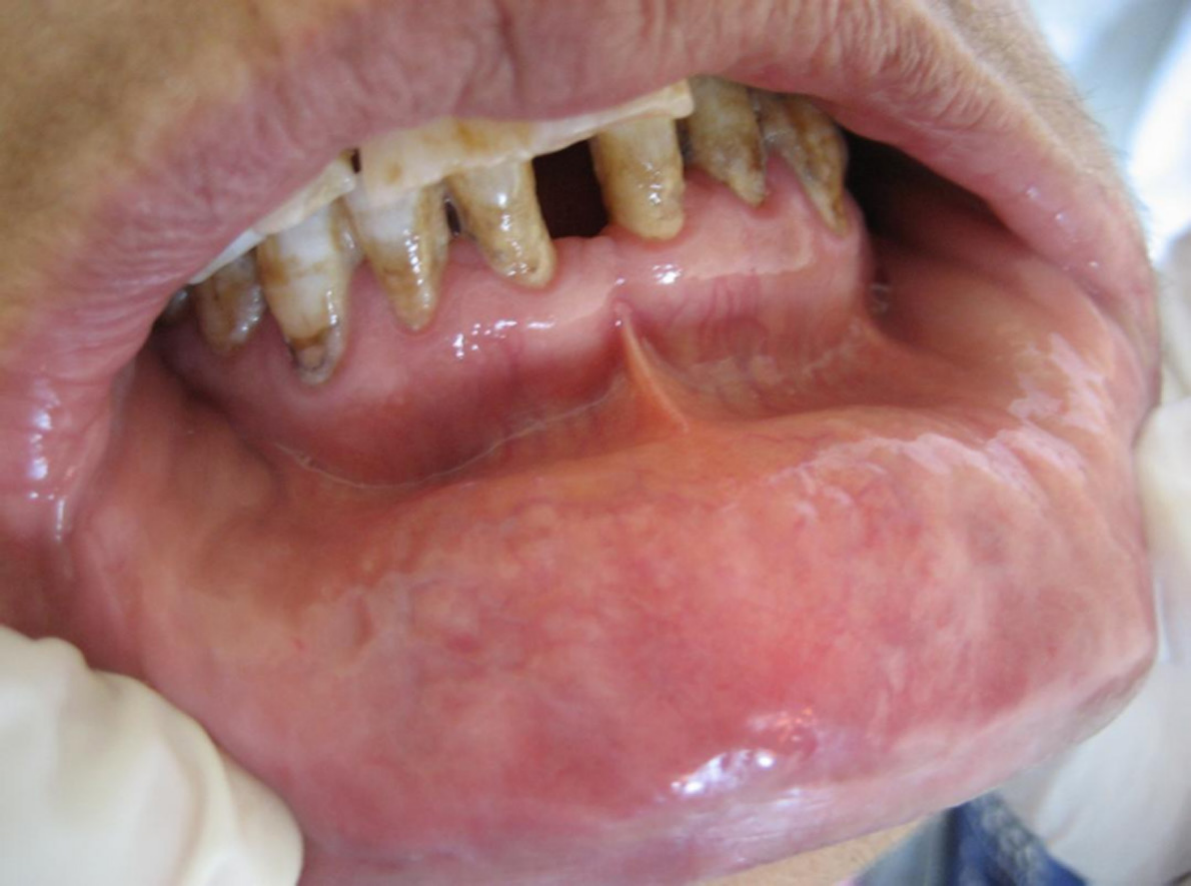
Complete remission of oral erythema multiforme lesions

**FIGURE 5:**
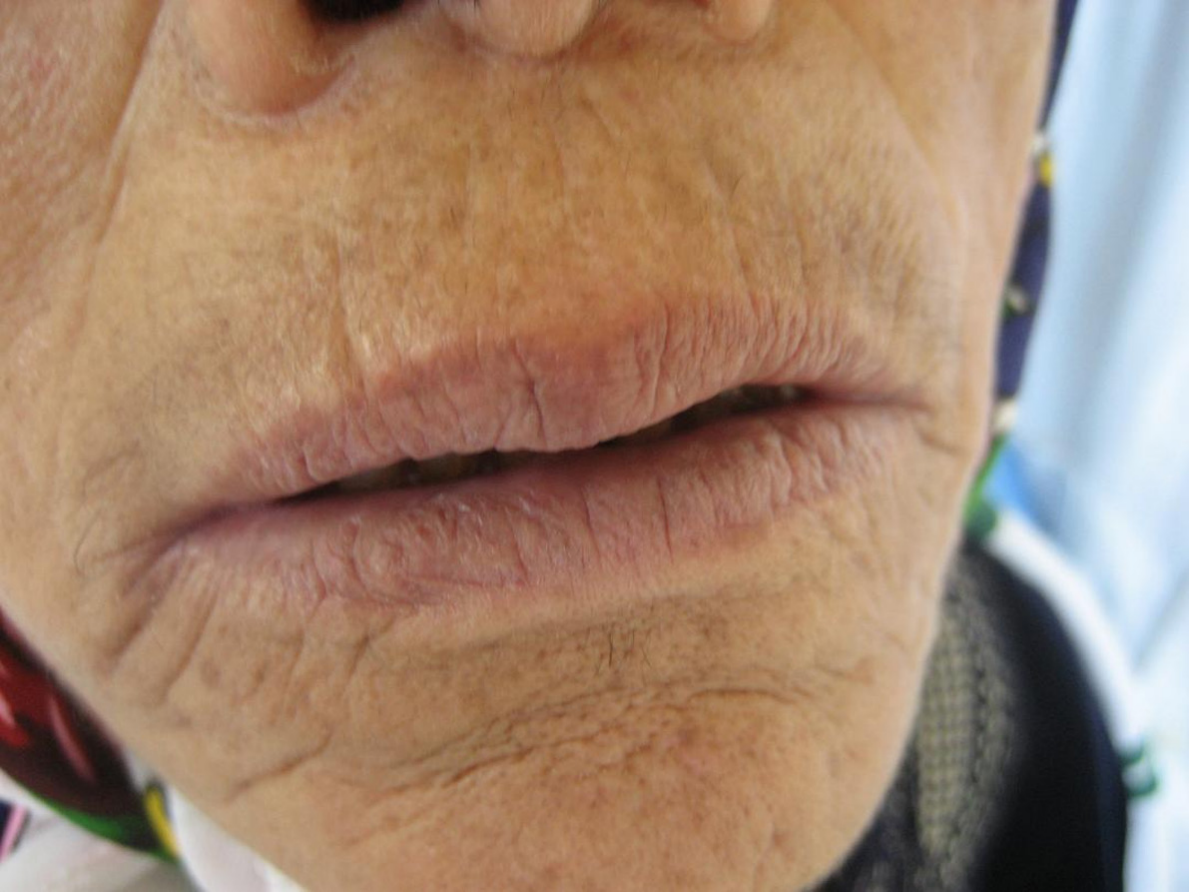
Complete remission of oral erythema multiforme lesions

A favorable healing was noticed within 7 days. At 1‐month follow‐up, the patient was completely asymptomatic, that confirmed our diagnosis (Figures 4 and 5).

## 3 DISCUSSION

Erythema multiforme is a reactive mucocutaneous disorder that was first described by Hebra in 1866^5^ affecting most often young adult people (20–40 years); however, it may involve children in 20% of cases.^4^, ^6^, ^7^ This inflammatory disorder is subclassified into three groups:

Erythema multiforme minor which was described by Hebra^6^ and characterized by ulcerations involving single mucosal site associated with typical target lesions.

Secondly, the group of erythema multiforme major to ulcerations involving more than one mucous membrane with also skin target lesions.^4^ Finally, for the lesions involving only the oral cavity, Kennett in 1968 reported the first case^8^; consequently, a third category known as oral erythema multiforme was described.^9^ In this last category, oral lesions have a predilection for the non‐keratinized mucosa and anterior part of the oral cavity, with this order of frequency: lips 36%, buccal mucosa 31%, tongue 22%, and labial mucosa 19%.^4^

More severe forms of erythema multiforme are described in 1922,^1^, ^7^, ^9^ such as Steven Johnson syndrome and toxic epidermal necrosis which are characterized by oral conjunctival mucous membrane and more extensive skin lesions.

Oral manifestations of erythema multiforme are characterized by large irregular erythematous ulcerations with or without pseudo membrane or nonspecific hyperkeratotic plaques, in addition to the presence of bullae or just erosions corresponding to broken blisters. As specific hue to oral erythema multiforme, we noted usually some hemorrhagic crusts in the upper and lower lip.^5^, ^9^, ^10^

For the etiology, erythema multiforme is usually related to an immunological reaction to bacterial (mycoplasma pneumonia, mycobacteria, brucellosis), viral (herpes simplex, AIDS, adenovirus, enterovirus), or fungal infections (coccidioidomycosis, dermatophytosis, histoplasmosis) infections, radiation therapy, malignancies, emotional stress, and drugs.as it was the case for our patient.^3^, ^4^, ^11^, ^12^ The most common drugs incriminated are as follows: sulfonamides, nonsteroidal anti‐inflammatory drugs, carbamazepine, vancomycin, phenytoin, co‐trimoxazole; barbituric….^9^, ^10^, ^13^, ^14^ Also, some bronchodilators and antiasthmatics can induce erythema multiforme as it was in our case in which we have the association between the bronchial asthma drug intake and incidence of lesions. Recently, Petruzzi et al described cases of oral erythema multiforme after Pfizer‐BioNTech COVID‐19 vaccination.^15^

The diagnosis of drug‐induced erythema multiforme is based on the pathognomonic clinical appearance and the distribution of the lesion, the positive drug history associated with the sudden onset of ulceration, and the exclusion of other inflammatory vesiculobullous lesions having similarities with this pathology, such as pemphigus vulgaris, bullous pemphigoid, bullous lichen planus, primary herpetic gingivostomatitis, as it was the case with our patient, likewise severe forms of drug reactions known as Steven Johnson syndrome and toxic epidermal necrosis.^5^, ^9^, ^10^ Distinguishing features must be noted for the differential diagnosis of erythema multiforme, such as Nikolsky sign and positive immunofluorescence for pemphigoid lesions,^1^, ^3^ the white reticular pattern for oral lichen planus,^16^ and the presence of atypical widespread skin and conjunctival lesions, for the severe forms.^2^, ^17^

The confirmation of diagnosis requires anatomopathological examination. Biopsy is advised in early vesicular lesions and not in ulcerated ones since histopathologic appearance is nonspecific.^10^

Histological features are characterized by perivascular mononuclear cell infiltrate and degenerative changes in the epithelium associated with intercellular or intracellular edema, acanthosis, and hyperkeratosis of the epithelium with hemorrhagic crusts.^9^

Management of oral erythema multiforme involves the elimination of the underlying causes, as it was the attitude with our patient by substitution of the drug. It requires also symptomatic treatment depending on the severity of lesions. It can be treated palliatively with analgesics for oral pain, soothing mouth rinses, viscous lidocaine rinse, soft liquid diet with avoidance of acidic, and spicy food. For secondarily infected lesions, systemic or topical antibiotics can be prescribed. Mostly, oral erythema multiforme lesions respond to topical steroids, and prescription of systemic ones is recommended for more severe cases.^9^, ^10^

## 4 CONCLUSION

Oral erythema multiforme is a benign reactive inflammatory lesion, presenting diverse etiologies that should be taken into account while treatment. It has characteristic clinical appearance that can be confused with many other diseases. Therefore, a prudent clinical examination must be performed in order to precisely determine the diagnosis and the adequate therapeutic approach.

## AUTHOR CONTRIBUTIONS

Maroua Garma: participate on carrying out the patient, redaction of the manuscript. Sameh Sioud: participate on making diagnosis and carrying out the patient, read and give final approval of the manuscript. Mounir Omami: read and approved the manuscript. Afef Slim: read and approved the manuscript. Chokri Abdellatif: read and approved the manuscript. Jamil Selmi: read and approved the manuscript.

## CONFLICT OF INTEREST

The authors declare that they have no conflict of interest.

## CONSENT

Written informed consent was obtained from the patient for his anonymized information to be published in this article.

## ETHICAL APPROVAL

Our institution does not require ethical approval for reporting individual cases or case series.

## Data Availability

The datasets generated during the current study are not publicly available but are available from the corresponding author on reasonable request.
